# Age‐Related Differences and Effects of Internalizing Symptoms on Aperiodic Neural Activity in Adolescents

**DOI:** 10.1111/psyp.70226

**Published:** 2026-01-03

**Authors:** Sarah E. Woronko, Angela Qian, Corinne N. Carlton, Ty Lees, Autumn Kujawa

**Affiliations:** ^1^ Department of Psychology and Human Development Vanderbilt University Nashville Tennessee USA; ^2^ Center for Anxiety, Depression, and Stress Research McLean Hospital Belmont Massachusetts USA; ^3^ Department of Psychiatry Harvard Medical School Boston Massachusetts USA

**Keywords:** 1/f, adolescence, anxiety, aperiodic activity, depression, EEG

## Abstract

The power spectrum derived from electroencephalography (EEG) recordings has been shown to include both periodic (e.g., delta, theta, alpha, beta) and aperiodic (e.g., 1/f exponent shape, vertical offset) components. Although internalizing disorders have been characterized by alterations in several periodic EEG measures, aperiodic activity has typically been treated as noise and unexamined in clinical neuroscience. Importantly, recent evidence has shown that aperiodic activity may reflect fundamental biological constructs such as excitatory‐inhibitory (E‐I) neural balance and neuronal cell spiking which are implicated in internalizing disorders. While previous research has found some evidence for blunted aperiodic parameters in adults with depression, few studies have probed the association between anxiety symptoms and aperiodic activity, and no work has probed these associations in adolescence when both mood and anxiety disorders commonly onset. Here, adolescents with current depressive disorders (*n* = 53), at high risk for depression due to maternal depressive history (*n* = 49), and at low risk for depression due to no maternal or personal depressive history (*n* = 51) completed an eyes‐closed resting state EEG paradigm to estimate aperiodic parameters. No group differences in aperiodic activity were observed, but age‐related declines in aperiodic exponent were moderated by depressive diagnosis such that those with a current depressive disorder diagnosis showed a steeper decline in the aperiodic exponent with age. Further, age‐related declines in aperiodic activity were moderated in opposite directions by internalizing symptoms such that elevated depressive symptoms steepened the inverse association between aperiodic activity and age, while elevated anxiety symptoms weakened this association. Results suggest unique alterations of aperiodic activity with internalizing symptoms and highlight the importance of considering aperiodic activity as a unique construct from periodic activity in investigating internalizing symptoms and brain development.

## Abstract 2

Aperiodic neural activity has historically been treated as noise despite its associations with excitatory‐inhibitory neural balance and neuronal cell spiking implicated across development and depressive disorders. This study is among the first to examine associations between aperiodic activity and internalizing symptoms and disorders in adolescents, finding evidence of interactions between age and depressive and anxiety symptoms, and highlighting the need to consider aperiodic EEG activity in clinical neuroscience.

## 1 Introduction

Electroencephalography (EEG) is commonly applied to measure periodic activity (i.e., oscillatory: theta, delta, alpha, beta) to study both psychopathology and development (Newson and Thiagarajan 2019). However, recent advances in EEG research have underscored the potential biological salience of irregular, non‐rhythmic aperiodic activity as captured by the 1/f shape of the spectrogram curve, which has previously been treated as noise (Donoghue et al. 2020). Aperiodic activity includes aperiodic offset (i.e., the horizontal shift of the 1/f curve) and the aperiodic exponent (i.e., the shape of the 1/f curve) which are theorized to relate to neuronal cell spiking and excitatory‐inhibitory (E‐I) balance, respectively (Donoghue et al. 2020; Gao et al. 2017; Manning et al. 2009). This supports the importance of examining aperiodic activity for both its effect on estimations of periodic activity and its biological relevance as a construct distinct from canonical periodic activity. Importantly, aperiodic activity is altered across various psychopathologies including depression (see Donoghue 2024 for a review) and is known to decrease across development through adulthood (Clark et al. 2024; Hill et al. 2022; Voytek et al. 2015). Specifically, both aperiodic offset and exponent were lower for adults with depression as compared to controls (Woronko et al. 2025) and increased following depression treatment (Smith et al. 2023; Veerakumar et al. 2019). While previous research has highlighted aperiodic activity as relevant to adult depression (Woronko et al. 2025; Rosenblum et al. 2023), it remains unclear how this presents in adolescence; a period of increased neuroplasticity as well as depression and anxiety incidence (Substance Abuse and Mental Health Services Administration 2022; Daly 2022; Avenevoli et al. 2015; Merikangas et al. 2010). Thus, in the present study we sought to examine the associations between adolescent depression and aperiodic parameters, as well as the extent to which these associations track with symptom severity, anxiety symptoms, and age.

Adolescence is characterized by high levels of neuroplasticity (Spear 2013) and is a sensitive period for the effects of risk factors of psychopathology (e.g., early adversity; Ho and King 2021; Sisk and Gee 2025). As such, adolescence is a crucial developmental period for studying individual differences in neural mechanisms that relate to cognitive and clinical risk factors including aperiodic activity (Donoghue 2024; Euler et al. 2024; McKeon et al. 2024). Similar to age‐related declines in aperiodic activity in adulthood, research on typical development in adolescents and young adults has highlighted age‐related decreases in aperiodic parameters cross‐sectionally (e.g., Hill et al. 2022; Angulo‐Ruiz et al. 2025) and longitudinally (e.g., McKeon et al. 2024; McSweeney et al. 2021). Age‐related decreases in aperiodic parameters can be at least partially explained by the neural noise hypothesis which posits that aging promotes increases in asynchronous neuronal firing leading to lower aperiodic offset and increases in relative excitation (i.e., faster decay of signal) to inhibition resulting in lower exponent values (Voytek et al. 2015). In fact, the predominant theory for aperiodic exponent is its representation of E‐I balance such that lower (flatter) exponent values reflect higher relative excitatory to inhibitory currents (Donoghue et al. 2020). In adolescence, magnetic resonance spectroscopy methods provide further support for the association between E‐I balance and aperiodic exponent. For example, age‐related decreases in aperiodic exponent have been associated with changes in the glutamate (excitatory neurotransmitter) to Gamma‐Aminobutyric Acid (GABA; inhibitory neurotransmitter) ratio (McKeon et al. 2024). Models of neuronal local‐field potentials have similarly supported aperiodic exponent as a proxy for E‐I balance (Gao et al. 2017; Manning et al. 2009).

In addition to typical development changes, alterations in aperiodic activity may be an important neurobiological factor underlying psychopathology (see Donoghue 2024 for a review). Given the theorized links between aperiodic offset and exponent to neuronal cell firing and E‐I balance, respectively, aperiodic activity may be particularly altered in psychopathologies characterized by E‐I mechanism deficits such as depression (Duman et al. 2019; Ironside et al. 2021, 2024; Moriguchi et al. 2019; Schür et al. 2016). To date, only a few studies have compared aperiodic activity in adults with and without depression (Woronko et al. 2025; Rosenblum et al. 2023; Li et al. 2025; Stolz et al. 2023; Zandbagleh et al. 2024), and findings are mixed. Stolz et al. (2023) and Li et al. (2025) found no group differences in aperiodic exponent for adults with major depressive disorder (MDD) and controls. In contrast, Zandbagleh et al. (2024) and Woronko et al. (2025) found decreased aperiodic parameters in a heterogeneous sample of individuals with MDD compared to controls, the latter effect of which was driven by increasing number of depressive episodes. These findings are further supported by treatment studies showing increases in aperiodic exponent following treatment (Smith et al. 2023; Veerakumar et al. 2019). Given that depression often onsets during adolescence (Substance Abuse and Mental Health Services Administration 2022; Daly 2022; Avenevoli et al. 2015), this developmental period is particularly important for identifying neural mechanisms of depression. However, no studies to date have examined aperiodic activity in adolescents with and without depressive disorders.

Though anxiety is highly comorbid with depression and also characterized by changes in E‐I modulatory mechanisms (Nuss 2015; Nasir et al. 2020), aperiodic activity has remained largely unstudied in anxiety disorders. In a recent review (Donoghue 2024), only four studies probing aperiodic activity within the context of anxiety and related disorders were identified. During resting state EEG for adults with post‐traumatic stress disorder, aperiodic activity was predictive of clinical status in a machine learning model (Li et al. 2022) and increased in individuals who responded to neuromodulation treatment (Makale et al. 2023). However, for awake adults with obsessive‐compulsive disorder (Perera et al. 2023) and asleep adults with heightened generalized anxiety (Blaskovich et al. 2024), no differences in aperiodic activity compared to controls were identified. Given such mixed and early results, more work is needed to understand how anxiety relates to aperiodic activity, especially in adolescence when anxiety disorders often onset (Merikangas et al. 2010) and given high rates of comorbidity between depression and anxiety.

Importantly, past work has highlighted associations between depression and an “aging brain” (Han et al. 2021; Dunlop et al. 2021). Han and colleagues (Han et al. 2021) found that, based on structural magnetic resonance imaging (MRI) scans, individuals with MDD were estimated to be older than their actual age. Similarly, Dunlop et al. (2021) found that, based on fMRI‐measured resting‐state functional connectivity estimates, individuals with MDD were estimated to be over 2 years older than their actual age. Together, these results support an association between depression and an accelerated aging brain, yet it has been understudied in adolescence, a period of high neuroplasticity and neurodevelopment. Such an association raises questions about how typical age‐related differences in aperiodic neural activity might be moderated by depression diagnoses or symptoms particularly in adolescence.

With this in mind, we sought to evaluate associations between adolescent depression and aperiodic parameters, as well as the extent to which these associations track with depressive symptoms, anxiety symptoms, and age. First, we examined group differences in aperiodic parameters for adolescents with current depressive disorders, adolescents with no history of depressive disorders but at elevated risk due to maternal history of depression, and adolescents with no personal or maternal history of depressive disorders. In line with patterns in adults (Woronko et al. 2025), we predicted that adolescents with depression would show decreased aperiodic parameters. Second, given strong evidence of an inverse association between age and aperiodic parameters (Hill et al. 2022; Voytek et al. 2015; McKeon et al. 2024), we examined the moderating effect of a current depressive disorder diagnosis on the association between age and aperiodic activity, predicting that adolescents with current depressive disorders would show steeper age‐related declines in aperiodic activity in line with the neural noise hypothesis of aging. To understand whether aperiodic activity tracks more with dimensional symptom severity rather than categorical measures of depression (i.e., depression diagnoses), we tested linear associations between depressive symptoms and aperiodic parameters and examined the moderating effect of depressive symptoms on the association between age and aperiodic activity. We similarly predicted that adolescents with high levels of depressive symptoms would show steeper age‐related declines in aperiodic activity than adolescents without depressive symptoms. Finally, we explored linear associations with anxiety symptoms and aperiodic activity, as well as interactions with age, which have not yet been studied in adults or adolescents.

## 2 Methods

### 2.1 Participants

Participants included 153 adolescents between the ages of 14 and 17 years (*M* = 15.20, SD = 1.08) separated into three groups based on their personal or maternal depression history: adolescents with current depressive disorders (CD, *n* = 53), adolescents with a high risk of depression due to maternal history of depression but no personal history of depression (HR, *n* = 49), and adolescents with a low risk of depression due to no maternal nor personal history of depression (LR, *n* = 51). Participant demographic information is displayed in Table 1. Inclusion criteria for the CD group required a current diagnosis of major depressive disorder (MDD), persistent depressive disorder (PDD), and/or unspecified depression for adolescents with clinically significant symptoms just short of symptom or duration criteria for a full MDD diagnosis. Of the 53 adolescents in this group, 16 (30.2%) met criteria for MDD only, 25 (47.2%) met criteria for PDD only, 11 (20.8%) met criteria for both PDD and MDD, and 1 (1.9%) met the predefined criteria for unspecified depression but not MDD or PDD. Adolescents who had been depressed in the past but not at study enrollment were not eligible.

**TABLE 1:** Demographic characteristics at screening of currently depressed, high risk, and low risk participants.

	CD	HR	LR	*F*/*χ* ^2^	*p*
	(*n* = 53)	(*n* = 49)	(*n* = 51)		
Demographics					
Female, *n* (%)	37 (69.81)	28 (57.14)	30 (58.82)	2.08	0.35
Age, years, mean (SD)	15.4 (1.10)	15.2 (1.09)	15.0 (1.04)	1.29	0.28
White, *n* (%)	36 (67.92)	36 (73.47)	36 (70.59)	22.36^c^	0.01
Black and/or African American, *n* (%)	9 (16.98)	10 (20.41)	5 (9.80)		
Asian, *n* (%)	0 (0)	1 (2.04)	8 (15.69)		
Native Hawaiian and/or Pacific Islander, *n* (%)	1 (1.89)	1 (2.04)	0 (0)		
American Indian and/or Alaska Native, *n* (%)	2 (3.77)	0 (0)	0 (0)		
Another race, *n* (%)	5 (9.43)	1 (2.04)	2 (3.92)		
Internalizing symptoms					
SCARED	36.3 (20.50)	24.2 (13.90)	16.7 (11.00)	19.11^a^	< 0.001
MFQ	28.3 (14.40)	10.4 (8.16)	7.59 (7.33)	56.90^a^	< 0.001
Cap montage and COVID timing					
Pre‐COVID‐19 enrollment, *n* (%)	28 (52.83)	14 (28.57)	19 (37.25)	6.47^b^	0.04
32 cap montage, *n* (%)	51 (96.22)	40 (81.63)	39 (76.47)	8.57^a^	0.01

Abbreviations: CD, current depressive disorder; HR, high risk for depression due to maternal history of depressive disorders; LR, low risk for depression due to no maternal history of depression; MFQ, Mood and Feelings Questionnaire; SCARED, Screen for Child Anxiety Related Disorders.

^a^ CD > HR and LR.

^b^ CD > HR only.

^c^ CD > LR only.

Adolescents were recruited via advertisements across Nashville and Vanderbilt University Medical Center. Adolescents in the CD group were also recruited through a separate depression treatment study that began enrollment earlier (Dickey et al. 2023), and EEG and clinical data were collected prior to treatment. Autism spectrum disorders, developmental disorders, intellectual disabilities, and personal or maternal history of mania or psychotic disorder were exclusionary. The three groups had different racial distributions (*p* = 0.013), although they were similar in age and sex distribution (*p*s ≥ 0.10; Table 1). Pairwise chi‐square tests showed that differences in racial distribution were between the CD and LR groups (*χ* ^2^ = 13.4, *p* = 0.020). Additionally, more participants had 32‐electrodes in the depressed group than the HR (*χ* ^2^ = 4.22, *p* = 0.040) and LR groups (*χ* ^2^ = 7.09, *p* = 0.008). Further, more depressed participants were enrolled pre‐COVID than participants in the HR (*χ* ^2^ = 5.23, *p* = 0.022) but not the LR (*χ* ^2^ = 1.96, *p* = 0.162) group.

### 2.2 Procedure

Study procedures were approved by the Vanderbilt University Institutional Review Board. Written assent from adolescents and written informed consent from their biological mothers were obtained prior to data collection. Semi‐structured clinical interviews of both the mother and adolescent were administered with a trained researcher to determine eligibility and group assignment. Mothers completed the Structured Clinical Interview for DSM‐5 (SCID; First 2015) to assess their lifetime history of depression and other psychopathology. Adolescent diagnoses were derived using the Kiddie Schedule of Affective Disorders and Schizophrenia (KSADS; Kaufman et al. 1997) administered to adolescents and mothers to assess lifetime history of psychopathology for the adolescent. Interviews were initially conducted in person and moved to Zoom during the COVID‐19 pandemic. A licensed clinical psychologist supervised interviews and reviewed all diagnoses. Independent raters reviewed and scored recordings, and interrater reliability was excellent (kappa = 1.0 for MDD and PDD).

Following the interviews, eligible participants completed the Mood and Feelings Questionnaire (Angold et al. 1995) and the Screen for Child Anxiety Related Disorders (Birmaher et al. 1997) (see the Methods in Appendix S1 for more information on each questionnaire) to assess depressive and anxious symptoms respectively, as well as a 6‐min resting state EEG assessment.

### 2.3 EEG Data Acquisition and Data Processing

Continuous EEG was recorded in a sound‐attenuated booth using a 32‐channel actiCHamp system in 10–20 configuration (Brain Products GmbH, Gilching, Germany). Study procedures were modified to reduce physical contact during the COVID‐19 pandemic such that for 23 participants (15.0%) only 16 channels were used. Data were recorded at a sampling rate of 1000 Hz and online referenced to Cz. Impedance values were maintained below 30 kΩ for all channels during collection. Three minutes of both eyes‐open and eyes‐closed data were collected in counterbalanced 1‐min trials. In line with previous research reporting no differences in aperiodic activity between eyes‐open and eyes‐closed data (Woronko et al. 2025) and demonstrating higher test–retest reliability for eyes closed trials than eyes open trials (Li et al. 2024), only eyes‐closed data were used in these analyses.

Data were preprocessed offline using BrainVision Analyzer (Version 2.3.0, Brain Products GmbH, Gilching, Germany). Data were IIR band pass filtered with cut‐offs at 0.1 Hz and 50 Hz (both with roll‐off order 2) and a 60 Hz notch filter. Next, data were re‐referenced to the average mastoid electrodes (TP9/TP10). Ocular artifacts were removed using Gratton and Cole's algorithm (Gratton et al. 1983). Artifact rejection was performed with semi‐automatic parameters removing datapoints meeting any of the following: voltage step greater than 50 μV/ms, voltage difference greater than 175 μV in a 400 ms interval, amplitude lower than −200 μV or greater than 200 μV, or activity lower than 0.5 μV in a 100 ms interval. Remaining artifacts were removed manually via visual inspection. Data were segmented into two‐second segments with one‐second overlap in line with Welch's Method (Welch 1967), omitting data with artifacts, after which segments were averaged, and power spectra were estimated for all channels using a fast Fourier transform. As recommended by Donoghue et al. (2020), participants with less than 60 segments used in averaging were excluded prior to analysis.

### 2.4 Neural Power Spectra Parameterization

**FIGURE 1:**
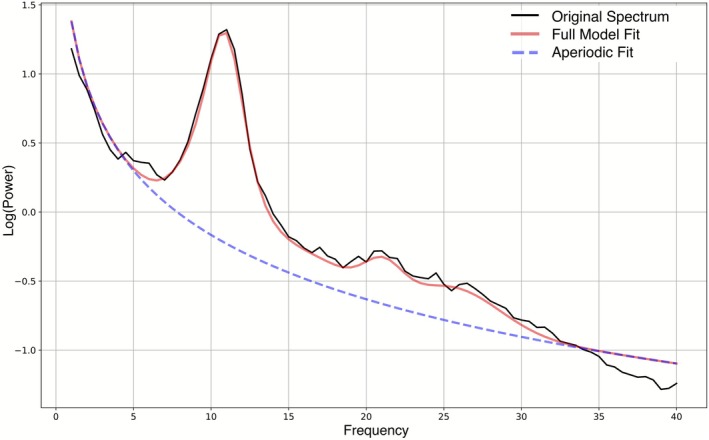
Example of a model fit at Cz for a representative participant with *R* ^2^ (0.99) and error (0.055) values representing the sample average model fit for Cz. The original power spectrum (i.e., 1/f‐curve; black) is plotted with the model fit (red). Aperiodic fit (blue) shows the 1/f shape curve of best fit to approximate the aperiodic exponent. In this image, aperiodic exponent is 1.55 and aperiodic offset is 1.38.

Exported power spectra were input into the FOOOF (v1.1.0) (Donoghue et al. 2020) pipeline to estimate aperiodic activity for each electrode for each participant with no fitted knee, using a frequency range of 1–40 Hz, minimum peak height of 0.05 μV, maximum peak width of 12, and maximum of 6 peaks. Alongside the aperiodic component estimates, the FOOOF pipeline also generates model fit parameters (*R* ^2^, error) for each estimated model. For this sample, average *R* ^2^ across all models was 0.983 ± 0.041, and average error was 0.050 ± 0.017. Average *R* ^2^ for Cz was 0.990 ± 0.006 and average error was 0.056 ± 0.017. These *goodness‐of‐fit* metrics were also used to remove any models with an *R* ^2^ less than 0.9 or error greater than 0.1 (128 models removed of 3728 models fit; 3.4%) prior to analysis. Figure 1 provides an example of the model, aperiodic component, and original data trace for one fitted model with representative mean error.

**FIGURE 2:**
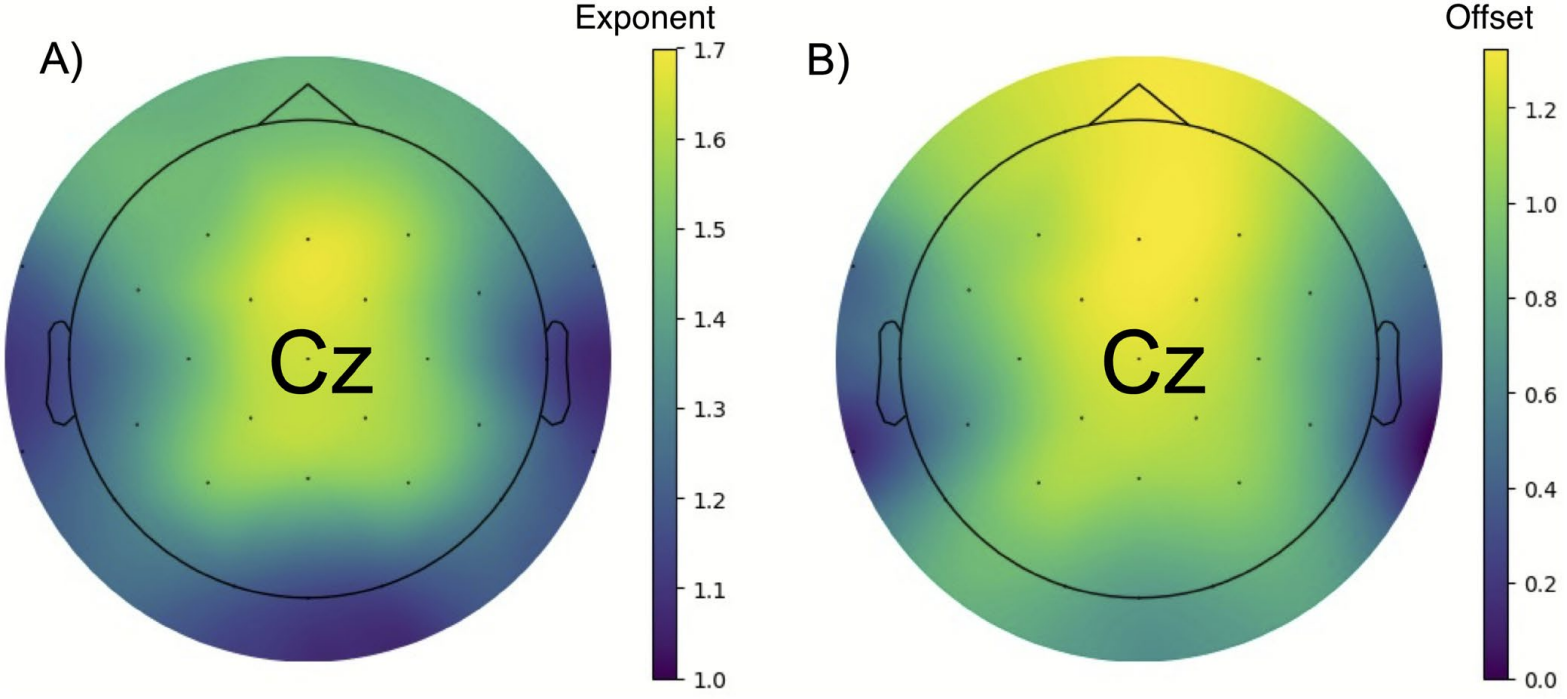
Scalp topography of sample exponent and offset values. Mean scalp topographies of aperiodic exponent (A; left) and offset (B; right). Center represents electrode Cz.

After removing bad fits, we then derived global averages for exponent and offset by taking the mean value from common electrodes on both the 16‐ and 32‐cap montage (i.e., Fz, Cz, Pz, Oz, O1, O2, CP1, CP2, FC1, FC2); ocular correction channels and reference channels were not included in this calculation. It is worth noting that any global average value calculated with more than one electrode missing (i.e., > 10% of data missing) was removed to prevent skewed estimation of the global average. Our analyses focused on global average aperiodic activity and aperiodic activity at Cz where it is typically maximal, in line with previous work (Donoghue et al. 2020); Figure 2 presents topographies of aperiodic exponent (A) and offset (B) magnitude.

### 2.5 Data Analysis Plan

All analyses were run in RStudio (v2024.9.1.394) (Posit Team 2024) and relevant packages are cited. We first conducted ANCOVA models controlling for age and number of channels in *rstatix* (Kassambara 2023), to test group differences (CD, HR, LR) in aperiodic parameters. Age is a robust, well‐established predictor of aperiodic activity (Voytek et al. 2015). By controlling for age, we better accounted for within‐group variance. Then, we used linear regression models to probe the interaction between depression diagnosis and age on aperiodic parameters. Next, we tested linear associations between study variables using Pearson correlations. Finally, we conducted hierarchical linear regression to test the linear associations and interactions between anxiety symptoms, depressive symptoms, and age, in predicting aperiodic parameters while controlling for number of channels in the electrode montage. Anxiety and depressive symptoms were included in the same model given past evidence that anxiety and depression often relate to neural indices in unique, opposing patterns that are suppressed if not controlled for in the same model (Kujawa et al. 2015; Weinberg et al. 2016). By including both anxiety and depression in the same model, we can elucidate these opposing effects. To compare how effects differed when depressive and anxiety symptoms were tested in separate models, we also present these models in Results A: Appendix S1. Given that data collection overlapped with the COVID‐19 pandemic and electrode montage down sampling, exploratory analyses were run with timing of data collection relative to March 2020 as an additional moderator of the depressive symptoms and age, and anxiety symptoms and age interactions. Results of these analyses are included in Results B: Appendix S1.

#### 2.5.1 Data Missingness and Outlier Analysis

Of the 153 participants who completed the EEG, 5 participants were missing MFQ scores and 9 participants were missing SCARED scores. An additional 27 participants (17.6%) were excluded from analyses due to excessive noise in reference channels (*n* = 16), poor EEG data quality resulting in low segment counts (*n* = 5), interpolation of more than 20% of channels (*n* = 5), or missing stimulus markers (*n* = 1).

We first ran an outlier analysis by group to remove global average and Cz exponent and offset values that exceeded three standard deviations from the group mean (see Results C: in Appendix S1 for group‐level mean values). All data missingness was handled using full information maximum likelihood (FIML) via the *lavaan* package (Rosseel 2012) in RStudio. We present a replicate analysis using all models regardless of their fit parameters in Results D: Appendix S1.

#### 2.5.2 Variable Transformations

Both the MFQ and SCARED were tested for normality to test multivariate normality assumptions by FIML (Enders and Bandalos 2001). The MFQ, but not the SCARED, produced a right‐skewed distribution (skewness = 1.159). Thus, MFQ was square‐root transformed prior to analyses to be more consistent with the assumptions of linear regression analyses and FIML (see Figure S1 for Q‐Q plots). All predictors were grand‐mean centered prior to being entered into the models.

## 3 Results

### 3.1 Group Differences in Aperiodic Parameters

**FIGURE 3:**
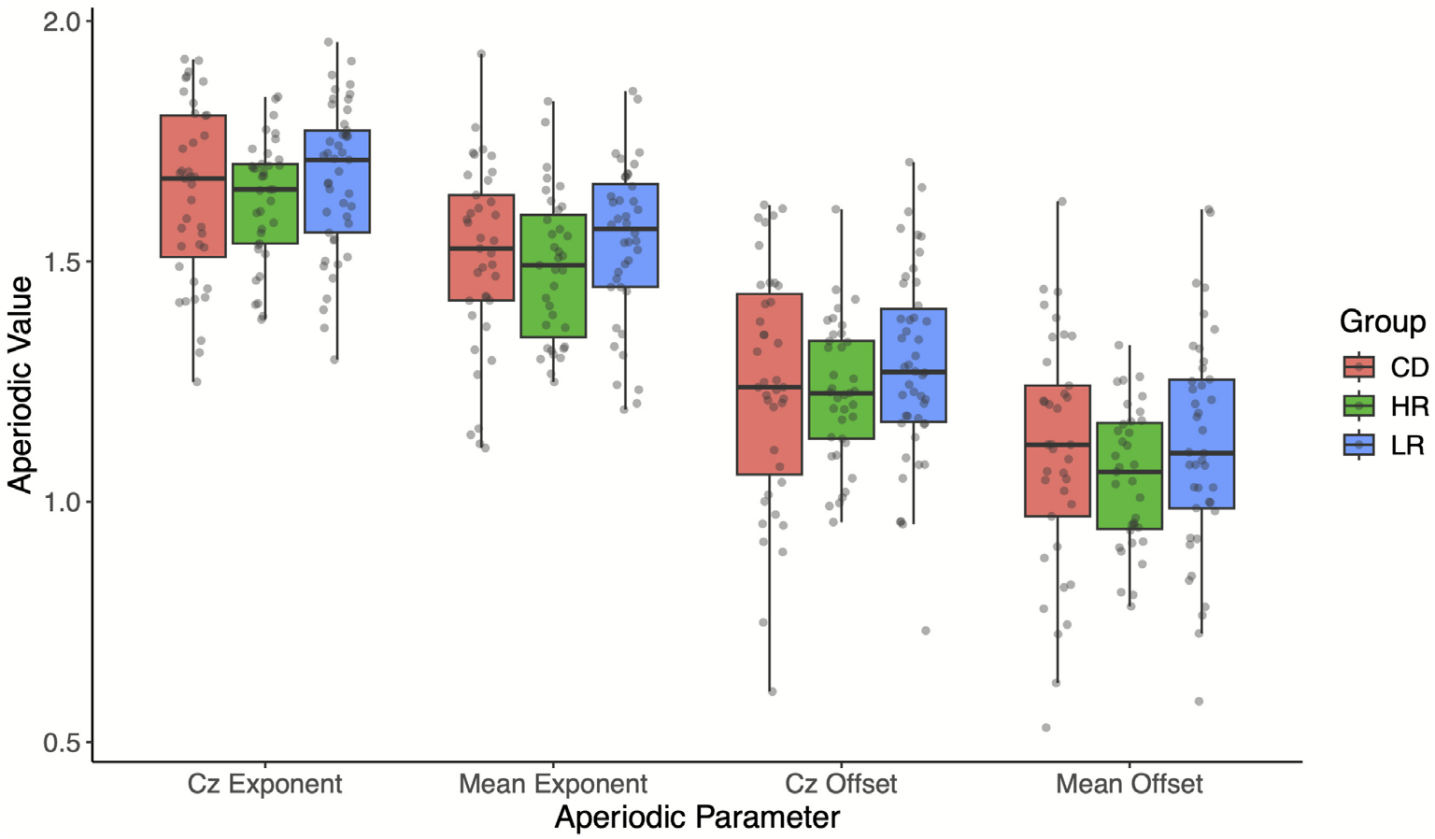
Group differences in aperiodic activity. Box plots with center line marking median, outer edges marking the 25th and 75th percentiles, and whiskers plotting ±1.5 times the interquartile range are provided for showing group distributions in aperiodic activity. CD, currently depressed; HR, high risk for depression due to maternal depressive history; LR, low risk for depression due to no maternal depressive history.

Group differences (CD, HR, LR) in aperiodic parameters were evaluated using ANCOVA models controlling for age and electrode montage. No significant differences between groups were observed for aperiodic exponent at Cz (*F*(2,116) = 0.493, *p* = 0.612, $\eta_{p}^{2}$ = 0.008), mean aperiodic exponent (*F*(2,107) = 0.863, *p* = 0.425, $\eta_{p}^{2}$ = 0.016), aperiodic offset at Cz (*F*(2,117) = 0.603, *p* = 0.549, $\eta_{p}^{2}$ = 0.010), or mean aperiodic offset (*F*(2,108) = 1.504, *p* = 0.227, $\eta_{p}^{2}$ = 0.027; Figure 3).

### 3.2 Depression Diagnosis by Age Effects for Aperiodic Parameters

**FIGURE 4:**
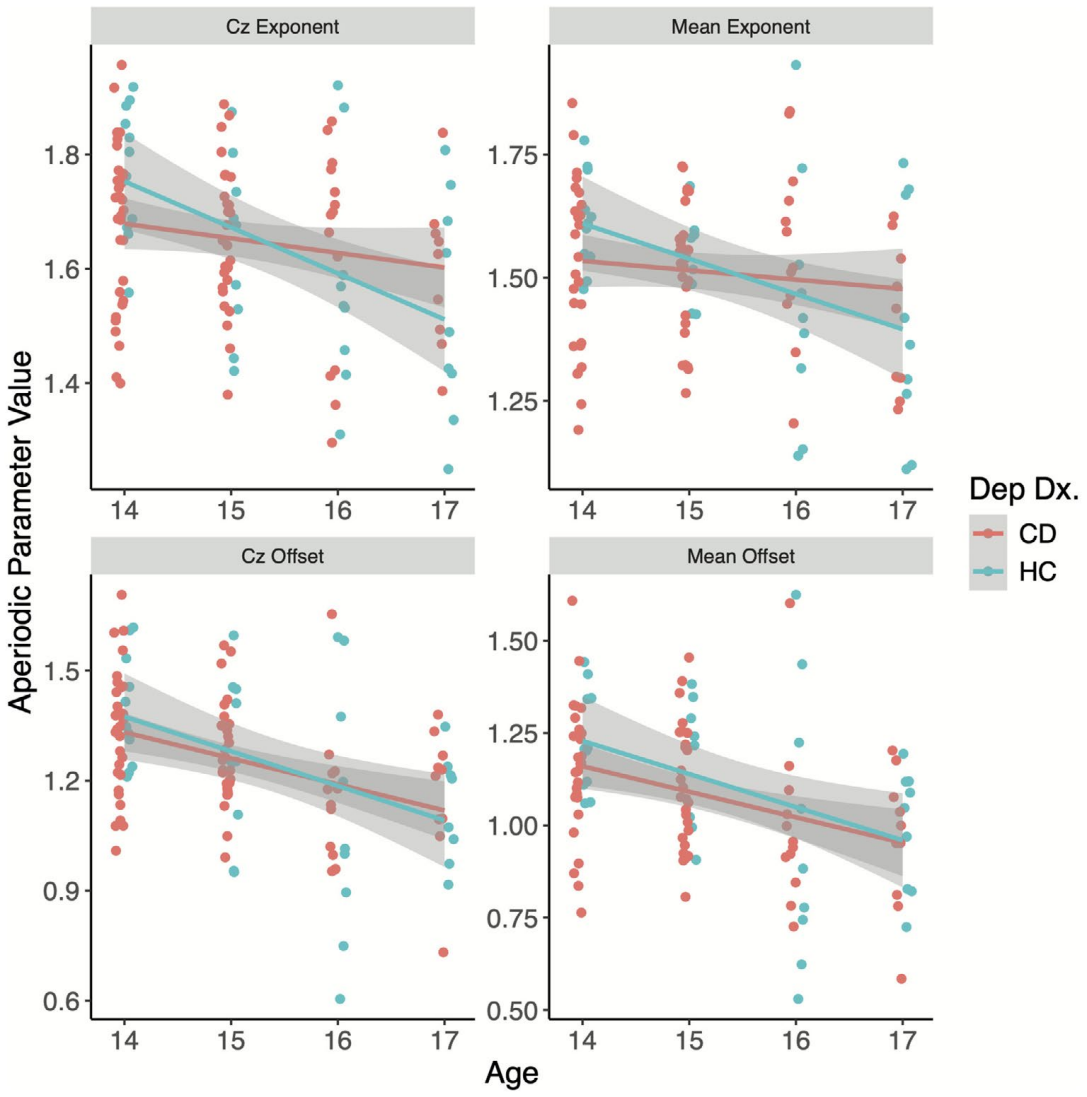
Differences in the negative relationship between age and aperiodic activity dependent on depressive disorder. Scatter plots are provided for showing the relationship between age and aperiodic activity for adolescents with and without depression. CD, currently depressed; Dx, depressive diagnosis; HC, never depressed adolescents (pooled high risk and low risk groups).

Given that no differences between HR and LR groups were identified and that both groups have no personal history of depressive disorders, we combined these groups into a non‐depressed control group to probe depression diagnosis by age interaction effects on aperiodic parameters. Supporting Information with comparisons between three groups are provided in Results E: Appendix S1. Regression models controlling for electrode montage revealed a significant interaction between age and depression diagnosis in the model predicting aperiodic exponent at Cz (*b =* −0.223, SE = 0.107, *p* = 0.037) such that the negative, linear association between age and aperiodic exponent was significant for CD adolescents (*b* = −0.080, *p* < 0.001) but not never depressed adolescents (*b* = −0.026, *p* = 0.116). The age by depression diagnosis interaction was marginally significant in the model predicting mean exponent, but not significant in models predicting offset (*p*s > 0.10; Table 2; Figure 4).

**TABLE 2:** Depression diagnosis by age effects for aperiodic parameters.

Aperiodic parameter ~	Regression statistics		
Model predictors	*β* (SE)	*p*	*R* ^2^
Exponent at Cz ~			
Electrodes	−0.046 (0.087)	0.593	0.128
Age	−0.176 (0.110)	0.110	
Dep. Dx.	0.033 (0.089)	0.712	
Dep. Dx. × Age	**−0.223 (0.107)**	**0**.**037**	
Mean Exponent ~			
Electrodes	**−0.194 (0.093)**	**0**.**037**	0.114
Age	−0.130 (0.116)	0.263	
Dep. Dx.	0.066 (0.093)	0.476	
Dep. Dx. × Age	−0.184 (0.111)	0.096	
Offset at Cz ~			
Electrodes	−0.049 (0.084)	0.559	0.179
Age	**−0.378 (0.100)**	**< 0.001**	
Dep. Dx.	0.043 (0.086)	0.618	
Dep. Dx. × Age	−0.072 (0.104)	0.491	
Mean Offset ~			
Electrodes	−0.088 (0.092)	0.338	0.157
Age	**−0.355 (0.196)**	**0**.**001**	
Dep. Dx.	0.112 (0.090)	0.211	
Dep. Dx. × Age	−0.057 (0.108)	0.594	

*Note:* A series of regression models were conducted to assess predictors of aperiodic parameters and regression statistics are provided. Full Information Maximum Likelihood was used to handle missing data. Dep. Dx. = depression diagnosis such that depressed adolescents (coded 1) and never depressed adolescents (pooled high risk and low risk groups; coded 0). Bold indicates *p* < 0.05.

### 3.3 Bivariate Associations With Age and Internalizing Symptoms

Pearson correlation analyses were used to examine the associations among aperiodic parameters, age, and anxiety and depressive symptoms. Aperiodic parameters showed significant positive associations with each other (*p*s < 0.01) and significant negative associations with age (*p*s < 0.01). Neither anxiety nor depression was correlated with age or aperiodic parameters (*p*s > 0.15; Table 3).

**TABLE 3:** Bivariate associations between study variables.

Variable	*M*	SD	1	2	3	4	5	6
1. Cz Exp	1.65	0.16						
2. Mean Exp	1.51	0.17	0.83**					
			[0.76, 0.88]					
3. Cz offset	1.25	0.20	0.70**	0.53**				
			[0.60, 0.78]	[0.38, 0.65]				
4. Mean offset	1.09	0.21	0.66**	0.67**	0.92**			
			[0.54, 0.75]	[0.56, 0.76]	[0.89, 0.95]			
5. Age	15.20	1.08	−0.31**	−0.24**	−0.41**	−0.38**		
			[−0.46, −0.14]	[−0.41, −0.06]	[−0.55, −0.25]	[−0.52, −0.21]		
6. SCARED	25.74	17.52	0.09	0.14	0.04	0.13	0.03	
			[−0.10, 0.27]	[−0.05, 0.33]	[−0.15, 0.22]	[−0.07, 0.31]	[−0.13, 0.20]	
7. MFQ	15.61	13.99	0.05	0.09	−0.06	0.02	0.10	0.76**
			[−0.14, 0.23]	[−0.11, 0.27]	[−0.24, 0.12]	[−0.17, 0.21]	[−0.07, 0.25]	[0.68, 0.82]

*Note:* Bivariate Pearson correlations between study variables provided. 95% confidence intervals presented in square brackets. Exp = Aperiodic exponent value; MFQ = Mood and Feelings Questionnaire (before square root transformation); SCARED = Screen for Child Anxiety Related Disorders.

** *p* < 0.01.

### 3.4 Depressive and Anxiety Symptoms by Age Effects for Aperiodic Parameters

In Step 1 of each hierarchical model, main effects of depressive and anxiety symptoms and covariates of participant age and number of electrodes were included to predict aperiodic exponent or offset at Cz and mean (*p*s > 0.10; Table 4). Step 2 of the hierarchical models added the interactions between depressive symptoms and age and anxiety symptoms and age. Interaction terms accounted for additional variance beyond the main effects (Δ*R* ^2^ = 0.046–0.053) in each model (Table 4).

**TABLE 4:** Internalizing symptoms and age as predictors of aperiodic parameters.

Regression statistics						
Aperiodic parameter ~	Step 1: Main effects			Step 2: Interaction model		
Model predictors	*β* (SE)	*p*	*R* ^2^	*β* (SE)	*p*	*R* ^2^
Exponent at Cz ~						
Electrodes	−0.065 (0.088)	0.459	0.110	−0.057 (0.085)	0.500	0.163
Age	**−0.322 (0.084)**	**< 0.001**		**−0.309 (0.082)**	**< 0.001**	
Anxiety Sx.	0.069 (0.133)	0.605		0.056 (0.129)	0.663	
Depressive Sx.	0.037 (0.133)	0.780		0.052 (0.129)	0.689	
Anxiety Sx. × Age	—	—		**0**.**279 (0.123)**	0.**023**	
Depressive Sx. × Age	—	—		**−0.298 (0.124)**	0.**016**	
Mean Exponent ~						
Electrodes	**−0.216 (0.092)**	**0.019**	0.119	**−0.210 (0.090)**	**0.019**	0.167
Age	**−0.260 (0.088)**	**0.003**		**−0.246 (0.086)**	**0.004**	
Anxiety Sx.	0.095 (0.135)	0.480		0.079 (0.131)	0.546	
Depressive Sx.	0.072 (0.136)	0.600		0.088 (0.133)	0.506	
Anxiety Sx. × Age	—	—		**0.249 (0.126)**	**0.048**	
Depressive Sx. × Age	—	—		**−0.283 (0.127)**	**0.025**	
Offset at Cz ~						
Electrodes	−0.053 (0.084)	0.533	0.177	−0.040 (0.082)	0.623	0.228
Age	**−0.411 (0.077)**	**< 0.001**		**−0.403 (0.075)**	**< 0.001**	
Anxiety Sx.	0.086 (0.130)	0.504		0.085 (0.126)	0.501	
Depressive Sx.	−0.062 (0.130)	0.631		−0.036 (0.126)	0.773	
Anxiety Sx. × Age	—	—		**0.313 (0.120)**	**0.009**	
Depressive Sx. × Age	—	—		−0.201 (0.121)	0.097	
Mean Offset ~						
Electrodes	−0.092 (0.092)	0.318	0.159	−0.079 (0.090)	0.377	0.205
Age	**−0.377 (0.081)**	**< 0.001**		**−0.370 (0.079)**	**< 0.001**	
Anxiety Sx.	0.145 (0.132)	0.273		0.142 (0.129)	0.271	
Depressive Sx.	−0.032 (0.134)	0.812		−0.004 (0.131)	0.974	
Anxiety Sx. × Age	—	—		**0.285 (0.123)**	**0.021**	
Depressive Sx. × Age	—	—		−0.176 (0.125)	0.159	

*Note:* A series of regression models were conducted to assess predictors of aperiodic parameters and regression statistics are provided. Full Information Maximum Likelihood was used to handle missing data. Depressive Sx. = Scores on the Mood and Feelings Questionnaire; Anxiety Sx. = Score on Screen for Child Anxiety Related Disorders; Bold indicates *p* < 0.05.

**FIGURE 5:**
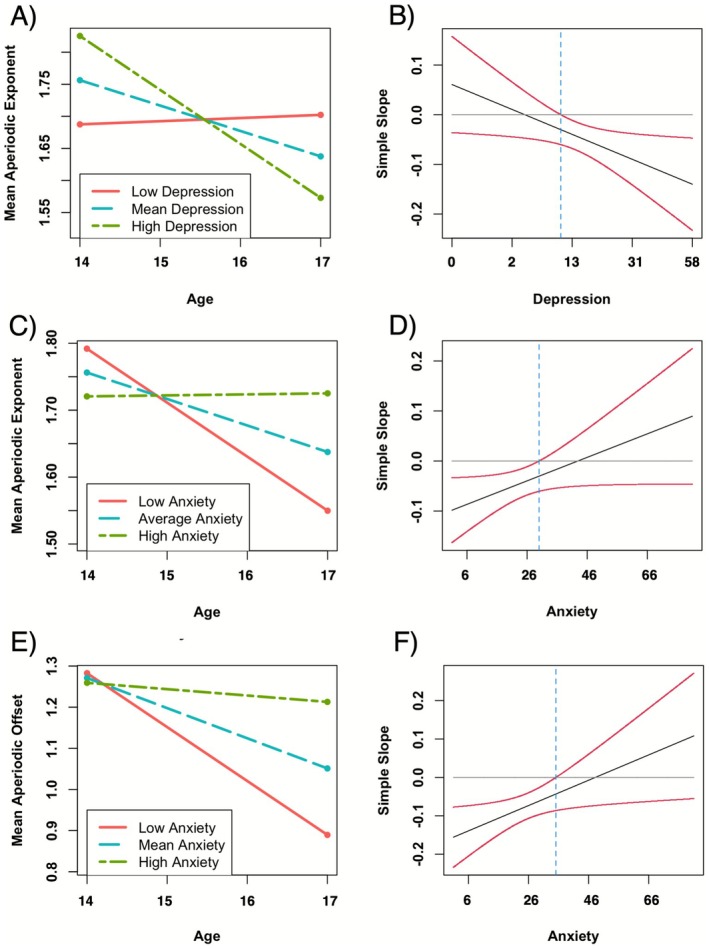
Simple slopes and Johnson‐Neyman output for interactions between age and both depressive and anxiety symptoms. Simple slopes and Johnson‐Neyman plots are provided for significant interactions. Depressive symptoms moderated the inverse association between age and aperiodic exponent (Plot A) such that age was inversely related to aperiodic exponent when MFQ score was greater than 8 (B). Anxiety symptoms moderated the inverse association between age and aperiodic exponent (Plot C) and offset (Plot E) such that age was inversely related to aperiodic parameters when SCARED score was less than 30 (Plot D) and 34 (Plot F), respectively. Since age, anxiety, and depressive symptom variables were centered and square‐root transformed (depressive symptoms only), *x*‐axis labels were plotted in the centered/square‐root ranges of the models, and tick marks were replaced with corresponding anchors in the dimensions of the scale for interpretation purposes.

Depressive symptoms moderated the inverse association between age and aperiodic exponent. Simple slopes analysis revealed that age and aperiodic exponent had the strongest inverse association when depressive symptoms were one standard deviation above the mean (approximately 28) (mean exponent: *b* = −0.084, SE = 0.024, *p* < 0.001; Cz exponent: *b* = −0.088, SE = 0.022, *p* < 0.001). This inverse association remained significant at the depressive symptom mean (approximately 13) for Cz (*b* = −0.045, SE = 0.013, *p* < 0.001) and mean exponent (*b* = −0.040, SE = 0.014, *p* = 0.006), but not for depressive symptoms one standard deviation below the sample mean (approximately 3; mean exponent: *b* = 0.005, SE = 0.026, *p* = 0.848; Cz exponent: *b* = −0.003, SE = 0.023, *p* = 0.912; Figure 5A). Age and aperiodic parameters were significantly negatively related when the square root, centered depressive symptom variable was greater than −0.377 (mean exponent) and −0.664 (Cz exponent), which approximates an MFQ score between 9 and 10 (Figure 5B).

Anxiety symptom scores were also a significant moderator of the inverse association between age and aperiodic parameters at Cz and mean aperiodic parameters. In contrast to depressive symptoms, simple slopes analysis revealed that age and aperiodic parameters had the strongest inverse association when anxiety symptoms were low (approximately 8) for Cz exponent (*b* = −0.088, SE = 0.022, *p* < 0.001), mean exponent (*b* = −0.081, SE = 0.025, *p* = 0.002), Cz offset (*b* = −0.137, SE *= *0.028, *p* < 0.001), and mean offset (*b* = −0.131, SE = 0.030, *p* < 0.001). This association remained significant at the anxiety symptom mean (approximately 25, indicative of the clinical anxiety cutoff on the SCARED; Caporino et al. 2017) for Cz exponent (*b* = −0.045, SE = 0.013, *p* < 0.001), mean exponent (*b* = −0.040, SE = 0.014, *p* = 0.006), Cz offset (*b* = −0.076, SE = 0.016, *p* < 0.001), and mean offset (*b* = −0.073, SE = 0.017, *p* < 0.001), but not for anxiety symptoms one standard deviation above the mean (*p*s > 0.10; approximately 43; Figure 5C,E). Age and aperiodic activity were significantly and inversely related when the centered anxiety symptom variable was below 9.11 for mean offset (approximately a score of 35), 10.04 for Cz offset (approximately a score of 36), 3.98 for mean exponent (approximately a score of 30), and 6.61 for Cz exponent (approximately a score of 32) (Figure 5D,F).

## 4 Discussion

We examined aperiodic activity in adolescents in association with depressive disorders and with symptoms of depression and anxiety. Contrary to hypotheses, aperiodic activity did not differ among groups, nor did aperiodic activity correlate with internalizing symptoms. However, in line with previous literature, aperiodic parameters were negatively associated with age. Interestingly, depression diagnosis moderated the inverse relationship between age and aperiodic exponent such that those with current depression showed steeper age‐related declines than never depressed adolescents. In addition, the inverse association between age and aperiodic exponent was moderated by both depressive and anxiety symptoms such that elevated depressive symptoms were related to steeper declines in aperiodic exponent with increasing age. In contrast, elevated anxiety symptoms were related to flatter declines in aperiodic exponent and offset with age.

Previous work comparing aperiodic parameters for adults with and without depression has shown mixed findings: some studies found reductions in aperiodic activity for individuals with current depressive disorders (Woronko et al. 2025; Rosenblum et al. 2023; Zandbagleh et al. 2024) while other studies found no differences similar to the present work (Li et al. 2025; Stolz et al. 2023). Methodological differences in resting‐state context (e.g., eyes‐open, eyes‐closed, sleep) and regions probed on the scalp (e.g., data‐driven, individual electrode, regional, global) could partially account for these conflicting findings. For example, previous work has shown that aperiodic activity differs across the scalp (Woronko et al. 2025). Another particularly compelling difference that could account for such mixed findings is the specificity of the depression group in each study. For example, Li et al. (2025) who found no differences in aperiodic parameters for individuals with and without MDD specifically recruited drug‐naïve individuals with MDD who were in their first episode of depression. In contrast, Woronko et al. (2025) observed lower aperiodic activity in a heterogeneous sample of adults with MDD compared to controls, an effect which was at least in part driven by the number of lifetime depressive episodes such that, similar to Li et al. (2025), individuals in their first episode did not differ from controls while individuals with five or more lifetime episodes had significantly lower aperiodic activity. In our sample of adolescents, group differences may be less apparent given that depression typically starts to emerge in this phase of life and extensive recurrences are not yet common. Thus, it may be the case that differences in aperiodic activity for individuals with and without depression only emerge for those who develop chronic, recurrent depression, which is not captured in a sample of adolescents, but more research is necessary to explore this hypothesis.

Importantly, to our knowledge, this is the first study to test both age‐related differences and internalizing symptom effects on aperiodic activity. Consistent with previous work (Clark et al. 2024; Hill et al. 2022; Voytek et al. 2015), we observed age‐related declines in aperiodic activity, but these age‐related differences were moderated by depressive and anxiety symptoms in unique ways. Our results suggest that depressive symptoms may steepen age‐related declines in aperiodic exponent, a result supported by other neurological evidence suggesting associations between depression and hastened neural aging (Han et al. 2021; Dunlop et al. 2021). Considering the aperiodic exponent as a proxy for E‐I balance, our findings further suggest that depressive symptoms relate to greater relative neural excitation which aligns with consistent patterns of blunted GABA signaling in individuals with depressive disorders (Duman et al. 2019; Ironside et al. 2024; Schür et al. 2016). In contrast, our results suggest that anxiety symptoms may flatten age‐related declines in aperiodic exponent, indicating higher inhibition relative to excitation. Indeed, both behavioral (Sandstrom et al. 2020) and neural over‐inhibition (Johnstone and Cohen Kadosh 2024) have been associated with heightened anxiety, though dysregulation in both excitatory and inhibitory systems has been implicated in internalizing symptoms and findings are often mixed (see Duman et al. 2019; Page and Coutellier 2019 for reviews). Future studies concurrently probing GABA and glutamate levels across neural regions and aperiodic activity as an E‐I balance proxy in individuals with and without internalizing symptoms will be necessary to substantiate these hypothesized associations.

Notably, electrode montage was associated with mean exponent for models without bad fits such that more electrodes in the montage (32‐electrodes) predicted lower exponent values, but not when including all models (Results D in Appendix S1). This result could be related to confounds in study design: electrode montage was downsized during the COVID‐19 pandemic, and the CD group was mostly recruited prior to the pandemic with a 32‐electrode montage. Thus, this finding may be driven by overrepresentation of the 32‐electrode montage in the CD group. Indeed, depressive disorders have been associated with lower exponent values (Woronko et al. 2025). Additionally, results from our exploratory analyses (see Results B in Appendix S1) including COVID‐19 timing in a three‐way interaction with anxiety or depressive symptoms and age showed that the anxiety and age interaction was significant for participants recruited before the pandemic, but not for participants recruited after the pandemic. Again, this effect may be driven by the CD group who was mostly recruited before the pandemic compared to the HR and LR groups. The onset of the COVID‐19 pandemic was an unexpected confound and important study limitation given that the CD group was recruited as part of a separate treatment study prior to the pandemic onset.

It is important to note that although depression diagnosis and age interaction effects on exponent were significant without accounting for anxiety symptoms, the depression and anxiety symptom interactions with age on exponent were only significant when tested in the same model (Results A in Appendix S1), though models with offset were consistent. When included in the same model, results indicate that elevated depressive symptoms steepen age‐related declines in aperiodic exponent while elevated anxiety symptoms blunt age‐related declines in aperiodic exponent. When separated, neither interaction reaches statistical significant, though the effect remains in the same direction. Past work has similarly found that anxiety and depression relate to neural indices in unique, opposing patterns that are suppressed if not included for in the same model (see Kujawa et al. 2015; Weinberg et al. 2016). Yet, these results should be interpreted cautiously given that symptoms of depression and anxiety are highly correlated with each other and interaction effects on exponent were not significant when tested in separate models. Future work is needed to compare individuals with both anxiety and depressive symptoms, depressive symptoms only, anxiety symptoms only, and neither anxiety nor depressive symptoms to clarify how internalizing symptoms elicit effects on aperiodic activity in adolescence.

A few additional limitations should be considered when interpreting our findings. First, our sample was recruited specifically based on current depressive disorders and risk for future episodes. This strategy excluded individuals who may have experienced a previous depressive episode but were remitted at the time of assessment. Further research probing the associations between depression and aperiodic parameters should consider how both current and past experiences of depression may alter aperiodic activity. Additionally, data was collected cross‐sectionally, limiting conclusions about developmental changes or the predictive utility of aperiodic parameters for future internalizing symptom development. Longitudinal data with broader age ranges will be important for tracking trajectories of typical and atypical development in aperiodic parameters, within‐person changes in aperiodic activity, and whether alterations in aperiodic activity are a risk factor for later internalizing symptoms. At a more granular level, EEG spatial resolution is limited, and activity at the electrode level reflects a summary of neural activity across the scalp. Thus, we cannot capture circuit or region specific changes with EEG, raising questions about the generalizability of findings to circuit‐ and region‐specific E‐I dysregulation (Duman et al. 2019; Page and Coutellier 2019). Future work conducting source‐related estimates of EEG activity and inclusion of multiple neuroimaging modalities will be important for continuing to probe the associations between aperiodic parameters and E‐I systems. Finally, to the best of our knowledge, this was the first study to probe the linear associations between internalizing symptomology and aperiodic activity in adolescence. While previous research has highlighted potential blunting effects of aperiodic parameters from depression (Rosenblum et al. 2023; Zandbagleh et al. 2024), few studies have explored associations between anxiety and aperiodic activity, underscoring the need for further studies to replicate the present findings. An important next step will be to recruit samples of individuals with and without anxiety and related disorders to compare aperiodic parameters.

In summary, while no group differences in aperiodic activity were shown for adolescents with and without current depressive disorders, depression diagnosis moderated the inverse relationship between age and aperiodic exponent such that those with current depression showed steeper age‐related declines in aperiodic exponent than never depressed adolescents. Interestingly, the age‐related declines in aperiodic activity were steepened by elevated depressive symptoms and flattened by elevated anxiety symptoms. Such findings extend the mixed findings comparing aperiodic activity in adults with and without depressive symptoms to adolescence and provide preliminary evidence for effects of elevated anxiety on aperiodic activity. Though resting state EEG has previously favored periodic activity, results underscore the importance of also considering aperiodic activity as an important construct for understanding internalizing symptoms and brain development.

## Author Contributions

**Sarah E. Woronko:** conceptualization, writing – original draft, methodology, visualization, writing – review and editing, formal analysis, data curation. **Angela Qian:** writing – review and editing, writing – original draft. **Corinne N. Carlton:** writing – review and editing. **Ty Lees:** methodology, writing – review and editing. **Autumn Kujawa:** conceptualization, methodology, resources, writing – review and editing, project administration, funding acquisition.

## Funding

This study was supported by the Klingenstein Third Generation Foundation Fellowship and Brain and Behavior Research Foundation Katherine Deschner Family Young Investigator Grant awarded to A. K. and the Vanderbilt Training Institute for Clinical and Translational Research UL1 TR000445 from NCATS/NIH; C. N. C. was supported by NIMH T32MH018921 and NIMH F32MH137993 during the completion of this work.

## Ethics Statement

Ethical approval for this study was obtained from the Vanderbilt University Institutional Review Board through protocol number 181983.

## Consent

Prior to participation, all participants provided informed consent in line with the protocols set by the Vanderbilt University Institutional Review Board and the Declaration of Helsinki.

## Conflicts of Interest

The authors declare no conflicts of interest.

## Supporting information


**Appendix S1:** Supporting Information.

## Data Availability

The data that support the findings of this study are available from the corresponding author upon reasonable request.
