# Supplementary material for: Age‐Related Differences and Effects of Internalizing Symptoms on Aperiodic Neural Activity in Adolescents
Source: Psychophysiology. 2026 Jan 3;63(1):e70226. doi: 10.1111/psyp.70226 (PMC12764435; doi:10.1111/psyp.70226)
Supplement: Supplementary file 1 — Appendix S1: Supporting Information. [file PSYP-63-e70226-s001.docx]

**Supplement Contents**

- **Supplemental Methods**
  - Questionnaires
  - *Supplemental Figure 1 - Q-Q plots before (left) and after (right) square root transform of Mood and Feelings Questionnaire.*
- **Supplemental Results**
  - Supplemental Results A – Symptom x Age models with anxiety and depressive symptoms tested in separate models
    - Supplemental Table A - *Internalizing symptoms and age as predictors of aperiodic parameters*
  - Supplemental Results B – COVID timing as a potential moderator of symptom by age interactions
    - *Supplemental Table B – Moderation effect of COVID timing on symptom by age interaction*
    - *Supplemental Figure B1 – Modeling significant three-way interaction between anxiety symptoms, age, and COVID timing predicting mean exponent (A) and main effect of COVID timing (B)*
  - Supplemental Results C – Group descriptive statistics
    - *Supplemental Table C - Group means and standard deviations for aperiodic parameters after outlier removal*
  - Supplemental Results D - All models included regardless of fit.
    - *Supplemental Table D1 - Group by age effects for aperiodic parameters (all models included)*
    - *Supplemental Table D2 - Internalizing symptoms and age as predictors of aperiodic parameters (all models included)*
  - Supplemental Results E – Group x Age effects for aperiodic parameters with all three groups.
    - *Supplemental Table E – Group by age effects for aperiodic parameters*
    - *Supplemental Figure E1 – Group by age for aperiodic parameters visualization*

**Supplemental Methods**

*Questionnaires*

*Mood and Feelings Questionnaire.* The 33-item version of the MFQ (37) was used to assess current depressive symptoms. Participants rated each item from 0 (*not true*) to 2 (*true*) to describe how they have been feeling or acting in the past two weeks, with higher scores indicating more severe depressive symptoms. The MFQ had excellent internal consistency in this sample (Cronbach’s alpha=.95).

*Screen for Child Anxiety Related Disorders.* The SCARED (38) consisted of 41 items to assess current anxiety symptoms. Adolescents rated each statement from 0 (*not true or hardly ever true*) to 2 (*very true or often true*) to describe their feelings over the past three months. Higher scores reflected higher severity of anxiety symptoms. Analyses focused on a total score to capture the heterogeneity of anxiety phenotypes. The SCARED had excellent internal consistency in this sample (Cronbach’s alpha=.96).

**Supplemental Figure 1:**

*Q-Q plots before (left) and after (right) square root transform of Mood and Feelings Questionnaire.*


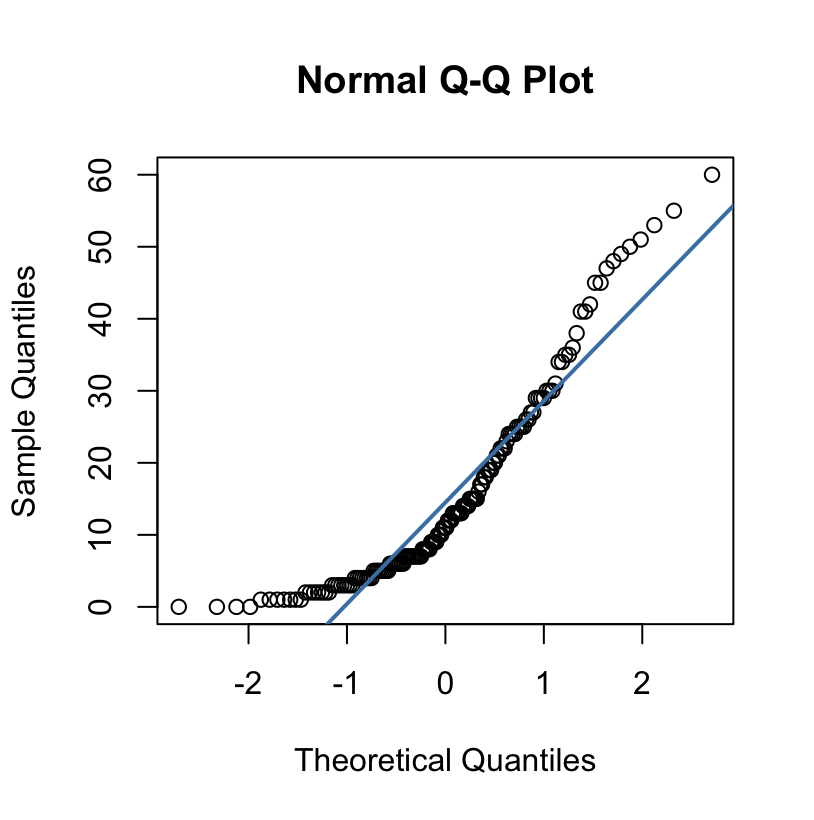

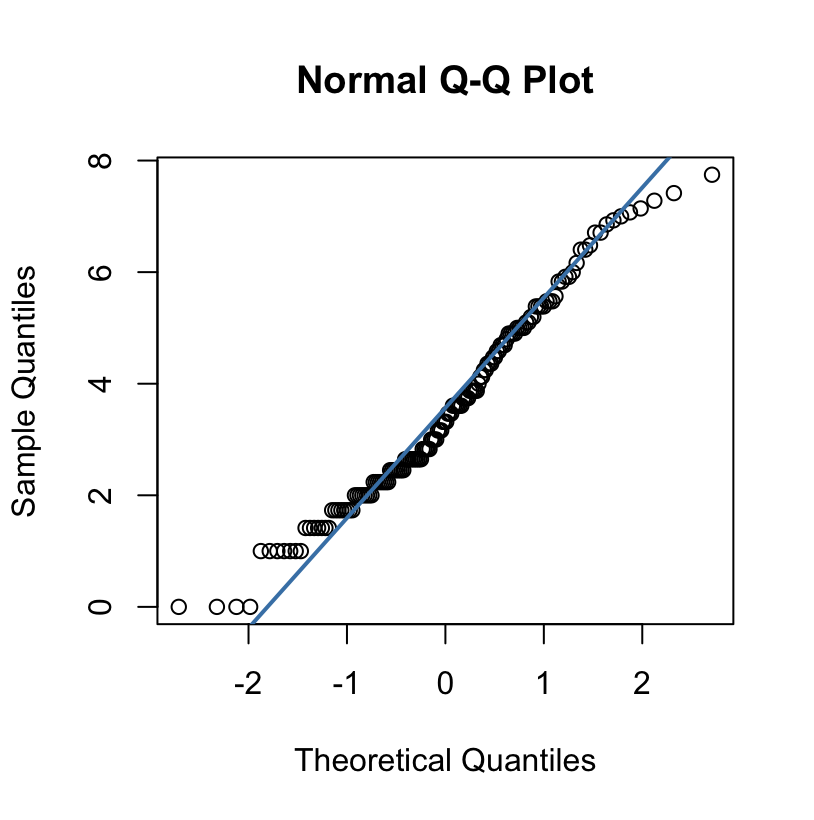


**Supplemental Results**

**Supplemental Results A – Symptom x Age models with anxiety and depressive symptoms tested in separate models**

Additional linear regression models were conducted with anxiety and depressive symptoms and their interaction with age separately modeled. In models predicting exponent, neither anxiety nor depression interacted with age to predict depressive symptoms (*p*s > .240). Similar to models containing both anxiety and depressive symptoms, age remained a significant, negative predictor of aperiodic exponent (*p*s < .004). Also, electrode montage remained a significant predictor of mean aperiodic exponent such that individuals with the 32-electrode montages had lower exponent values. In contrast to models containing both anxiety and depressive symptoms, anxiety symptoms were a significant predictor of mean aperiodic exponent such that increases in anxiety related to increases in mean exponent (*β =* .190, *p* = .040) but did not interact with age (*β* = .084, *p* = .367).

In models predicting offset, results parallel those reported in models containing both anxiety and depressive symptoms. Anxiety and age, but not depressive symptoms and age, interacted to predict aperiodic offset (Cz: *β =* .196, *p* = .025; Mean: *β =* .189, *p* = .033). Age remained a significant, negative predictor of aperiodic offset (*p*s < .001).

**Supplemental Table A - *Internalizing symptoms and age as predictors of aperiodic parameters***

|  | **Regression Statistics** | | | | | | | |
| --- | --- | --- | --- | --- | --- | --- | --- | --- |
| Aperiodic parameter ~ | | Depression Effects | | |  | Anxiety Effects | | |
| Model Predictors | | *β (SE)* | *p* | *R^2^* |  | *β (SE)* | *p* | *R^2^* |
| Exponent at Cz ~  Electrodes  Age  Dep Sx.  Dep Sx. x Age | | -.062 (.086)  **-.322 (.083)**  .076 (.090)  -.100 (.092) | .470  **<.001**  .403  .275 | .120 | Exponent at Cz ~  Electrodes  Age  Anx Sx.  Anx Sx. x Age | -.061 (.087)  **-.313 (.082)**  .122 (.092)  .099 (.092) | .486  **<.001**  .182  .280 | .121 |
| Mean Exponent ~  Electrodes  Age  Dep Sx.  Dep Sx. x Age | | **-.214 (.091)**  **-.259 (.087)**  .129 (.092)  -.109 (.093) | **.019**  **.003**  .163  .240 | .129 | Mean Exponent~  Electrodes  Age  Anx Sx.  Anx Sx. x Age | **-.213 (.092)**  **-.247 (.086)**  **.190 (.092)**  .084 (.093) | **.020**  **.004**  **.040**  .367 | .133 |
| Offset at Cz ~  Electrodes  Age  Dep Sx.  Dep Sx. x Age | | -.043 (.084)  **-.417 (.076)**  .002 (.090)  .016 (.090) | .605  **<.001**  .986  .864 | .174 | Offset at Cz ~  Electrodes  Age  Anx Sx.  Anx Sx. x Age | -.040 (.083)  **-.409 (.075)**  .081 (.089)  **.196 (.087)** | .632  **<.001**  .361  **.025** | .210 |
| Mean Offset ~  Electrodes  Age  Dep Sx.  Dep Sx. x Age | | -.080 (.092)  **-.386 (.080)**  .075 (.093)  .017 (.092) | .386  **<.001**  .418  .181 | .150 | Mean Offset ~  Electrodes  Age  Anx Sx.  Anx Sx. x Age | -.079 (.090)  **-.371 (.078)**  .174 (.090)  **.189 (.089)** | .382  **<.001**  .053  **.033** | .199 |

*Note.* A series of regression models were conducted to assess predictors of aperiodic parameters and regression statistics are provided. Full Information Maximum Likelihood was used to handle missing data. Dep Sx. = Scores on the Mood and Feelings Questionnaire; Anx Sx. = Score on Screen for Child Anxiety Related Disorders; Bold indicates *p* < .05.

**Supplemental Results B – COVID timing as a potential moderator of internalizing symptom by age interactions**

*COVID timing.* Linear regression models were repeated with inclusion of a three-way interaction term between COVID timing (individuals enrolled pre- vs post-COVID), symptoms, and age. Post-COVID onset was defined as data collection completed following March 2020. There was a significant three-way interaction between anxiety symptoms, age, and COVID timing in prediction mean exponent (*b*=-.322, *p*=.008), but no other significant three-way interaction emerged. Post hoc probes of the three-way interaction between COVID timing, anxiety symptoms, and age predicting mean exponent revealed that the two-way interaction between anxiety and age was significant for individuals’ pre-COVID (*b=*.597, *p*<.001), but not post-COVID (*b*=-.019, *p*=.912)*.* Additional exploratory comparisons between the sample pre- and post-COVID showed no significant differences in age (*F*(1,151)=1.27, *p*=.26), anxiety symptoms (*F*(1,142)=0.81, *p*=.37), or depressive symptoms (*F*(1,146)=3.34, *p*=.07).

**Supplemental Table B –** ***Moderation effect of COVID timing on symptom by age interaction***

| Regression Statistics | | | | |  |  |  |  |  |
| --- | --- | --- | --- | --- | --- | --- | --- | --- | --- |
| Aperiodic parameter ~ |  |  | | | Aperiodic parameter ~ |  |  |  |  |
| Model Predictors | *β (SE)* | | *p* | *R^2^* | Model Predictors | *β (SE)* | *p* | *R^2^* |  |
| Exponent at Cz ~  Electrodes  Age  COVID  Anxiety Sx.  Depressive Sx.  Anxiety Sx. x Age  Depressive Sx. x Age  COVID x Age  COVID x Anxiety Sx.  COVID x Dep. Sx.  Anx x Age x COVID  Dep x Age x COVID | -.092 (.087)  **-.388 (.090)**  -.061 (.091)  .034 (.131)  .067 (.132)  **.273 (.125)**  -.256 (.133)  .126 (.094)  .141 (.128)  -.100 (.130)  -.145 (.126)  .059 (.136) | | .290  **<.001**  .500  .793  .613  **.030**  .055  .178  .272  .445  .250  .666 | .200 | Offset at Cz ~  Electrodes  Age  COVID  Anxiety Sx.  Depressive Sx.  Anxiety Sx. x Age  Depressive Sx. x Age  COVID x Age  COVID x Anxiety Sx  COVID x Dep. Sx.  Anx x Age x COVID  Dep x Age x COVID | -.080 (.081)  **-.425 (.077)**  -.137 (.083)  .070 (.124)  -.030 (.123)  **.331 (.117)**  -.175 (.119)  .050 (.084)  .207 (.120)  **-.251 (.119)**  -.010 (.119)  -.077 (.122) | .329  **<.001**  .099  .573  .806  .**005**  .144  .548  .085  **.035**  .936  .524 | .297 |  |
| Mean Exponent ~  Electrodes  Age  COVID  Anxiety Sx.  Depressive Sx.  Anxiety Sx. x Age  Depressive Sx. x Age  COVID x Age  COVID x Anxiety Sx.  COVID x Dep. Sx.  Anx x Age x COVID  Dep x Age x COVID | **-.307 (.085)**  **-.236 (.093)**  **-.239 (.089)**  .115 (.129)  .043 (.132)  **.314 (.123)**  **-.307 (.131)**  .062 (.095)  .089 (.127)  -.008 (.131)  **-.322 (.122)**  .191 (.134) | | **<.001**  **.011**  **.007**  .373  .744  **.011**  **.019**  .512  .483  .949  **.008**  .154 | .285 | Mean Offset ~  Electrodes  Age  COVID  Anxiety Sx.  Depressive Sx.  Anxiety Sx. x Age  Depressive Sx. x Age  COVID x Age  COVID x Anxiety Sx  COVID x Dep. Sx.  Anx x Age x COVID  Dep x Age x COVID | -.164 (.085)  **-.399 (.079)**  **-.250 (.083)**  .150 (.124)  -.010 (.124)  **.343 (.116)**  -.171 (.118)  .038 (.085)  .224 (.120)  -.215 (.121)  -.152 (.119)  -.014 (.121) | .054  **<.001**  **.003**  .226  .935  **.003**  .148  .654  .063  .075  .202  .906 | .335 |  |

*Note.* A series of regression models were conducted to assess predictors of aperiodic parameters and regression statistics are provided. Full Information Maximum Likelihood was used to handle missing data. COVID was dummy coded such that pre-COVID enrollment = 1 and post-COVID enrollment = -1. Dep = Depressive Sx. = Scores on the Mood and Feelings Questionnaire; Anx = Anxiety Sx. = Score on Screen for Child Anxiety Related Disorders; Bold indicates *p* < .05.

*Supplemental Figure B1* ***– Modeling significant three-way interaction between anxiety symptoms, age, and COVID timing predicting mean exponent (A) and main effect of COVID timing (B)***

(A)

(B)

**Supplemental Results C – Group descriptive statistics**

*Supplemental Table C - Group means and standard deviations for aperiodic parameters after outlier removal*

|  | CD  (*n*=53) | HR  (*n*=49) | LR  (*n*=51) |
| --- | --- | --- | --- |
| **Exponent** |  |  |  |
| Cz | 1.64 (.19) | 1.63 (.13) | 1.67 (.16) |
| Mean | 1.51 (.20) | 1.49 (.15) | 1.54 (.17) |
| **Offset** |  |  |  |
| Cz | 1.24 (.25) | 1.23 (.15) | 1.28 (.20) |
| Mean | 1.10 (.25) | 1.05 (.14) | 1.12 (.23) |

*Note.* CD = Currently depressed; HR = High risk for depression due to maternal depressive history; LR = Low risk for depression due to not maternal or personal history of depression

**Supplemental Results D - All models included regardless of fit.**

*Group differences in aperiodic parameters (all models included)*

To test group differences (CD, HR, LR) in aperiodic parameters, regardless of model fit (i.e., *R*^2^ values of any magnitude), a series of ANCOVA models controlling for age and electrode montage were conducted. No significant differences between groups were observed for aperiodic exponent at Cz (*F*(2, 119) = .242, *p* = .786, *ηp^2^* = .004), mean aperiodic exponent (*F*(2, 119) = .567, *p* = .569, *ηp^2^* = .009), aperiodic offset at Cz (*F*(2, 119) = .561, *p* = .572, *ηp^2^* = .009), or mean aperiodic offset (*F*(2, 119) = 1.433, *p* = .243, *ηp^2^* = .024).

*Group by age effects for aperiodic parameters (all models included)*

Given that no differences between HR and LR groups were identified, we combined these groups into a non-depressed control group to probe group by age interaction effects on aperiodic parameters with inclusion of all models regardless of error and R^2^ value. Regression models controlling for electrode montage revealed a marginally significant interaction between age and group in the model predicting aperiodic exponent at Cz, but no significant group by age interaction in models predicting offset or mean exponent (*p*s>.10).

**Supplemental Table D1 - *Group by age effects for aperiodic parameters (all models included)***

| Aperiodic parameter ~ | Regression Statistics | | |
| --- | --- | --- | --- |
| Model Predictors | *R^2^* | *β (SE)* | *p* |
| Exponent at Cz ~  Electrodes  Age  Group  Group x Age | .133 | -.035 (.085)  **-.374 (.083)**  .023 (.089)  -.169 (.088) | .678  **<.001**  .799  .055 |
| Mean Exponent ~  Electrodes  Age  Group  Group x Age | .079 | -.050 (.087)  **-.292 (.088)**  .047 (.091)  -.066 (.092) | .570  **.001**  .610  .470 |
| Offset at Cz ~  Electrodes  Age  Group  Group x Age | .183 | -.043 (.082)  **-.442 (.078)**  .044 (.086)  -.058 (.086) | .602  **<.001**  .609  .498 |
| Mean Offset ~  Electrodes  Age  Group  Group x Age | .174 | -.056 (.083)  **-.413 (.079)**  .093 (.086)  .016 (.087) | .497  **<.001**  .281  .855 |

*Note.* A series of regression models were conducted to assess predictors of aperiodic parameters and regression statistics are provided. Full Information Maximum Likelihood was used to handle missing data. Group = depressed adolescents and never depressed adolescents (pooled high risk and low risk groups).

*Internalizing symptoms and age as predictors of aperiodic parameters*

Hierarchical linear regression models were repeated with inclusion of all models regardless of error and *R*^2^ value. The only models that were excluded were those with values exceeding 3 standard deviations from the mean. The results of these models are tabulated below.

**Supplemental Table D2 -** ***Internalizing symptoms and age as predictors of aperiodic parameters (all models included)***

|  | Regression Statistics | | | | | | | | | | | | |  |
| --- | --- | --- | --- | --- | --- | --- | --- | --- | --- | --- | --- | --- | --- | --- |
| Aperiodic parameter ~ |  | Step 1: Main Effects | | | |  |  | | Step 2: Interaction Model | | | | |  |
| Model Predictors | *R^2^* | | *β (SE)* | | *p* | | | *R^2^* | | | *β (SE)* | | *p* |  |
| Exponent at Cz ~  Electrodes  Age  Anxiety Sx.  Depressive Sx.  Anxiety Sx. x Age  Depressive Sx. x Age | .117 | | | -.056 (.085)  **-.337 (.082)**  .060 (.132)  .039 (.132)  --  -- | .511  **<.001**  .650  .765  --  -- | | | .170 | | -.047 (.083)  **-.324 (.081)**  .047 (.128)  .055 (.128)  **.282 (.122)**  **-.294 (.124)** | | .571  **<.001**  .711  .667  **.021**  **.017** | |  |
| Mean Exponent ~  Electrodes  Age  Anxiety Sx.  Depressive Sx.  Anxiety Sx. x Age  Depressive Sx. x Age | .089 | | | -.068 (.086)  **-.284 (.085)**  .037 (.133)  .089 (.133)  --  -- | .472  **.001**  .778  .506  --  -- | | | .118 | | -.055 (.085)  **-.276 (.084)**  .031 (.131)  .102 (.131)  .218 (.127)  -.199 (.129) | | .520  **.001**  .813  .439  .085  .122 | |  |
| Offset at Cz ~  Electrodes  Age  Anxiety Sx.  Depressive Sx.  Anxiety Sx. x Age  Depressive Sx. x Age | .180 | | | -.046 (.082)  **-.416 (.076)**  .085 (.129)  -.059 (.129)  --  -- | .578  **<.001**  .513  .648  --  -- | | | .232 | | -.034 (.080)  **-.408 (.074)**  .083 (.125)  -.034 (.125)  **.315 (.119)**  -.200 (.121) | | .670  **<.001**  .508  .788  **.008**  .098 | |  |
| Mean Offset ~  Electrodes  Age  Anxiety Sx.  Depressive Sx.  Anxiety Sx. x Age  Depressive Sx. x Age | .174 | | | -.056 (.082)  **-.409 (.076)**  .095 (.128)  .010 (.128)  --  -- | .495  **<.001**  .458  .938  --  -- | | | .214 | | -.046 (.081)  **-.403 (.075)**  .097 (.125)  .030 (.125)  **.267 (.121)**  -.142 (.122) | | .572  **<.001**  .439  .808  **.027**  .245 | |  |

*Note.* A series of regression models were conducted to assess predictors of aperiodic parameters and regression statistics are provided. Full Information Maximum Likelihood was used to handle missing data. Depressive Sx. = Scores on the Mood and Feelings Questionnaire; Anxiety Sx. = Score on Screen for Child Anxiety Related Disorders; Bold indicates *p* < .05.

**Supplemental Results E – Group x Age effects for aperiodic parameters with all three groups.**

We probed group by age interaction effects on aperiodic parameters while keeping the HR and LR groups separate. Regression models controlling for electrode montage with depressed group as the reference revealed a significant interaction between age and the HR group in the model predicting aperiodic exponent at Cz (*b*=.260, *SE*=.121, *p*=.032), but not significant in models predicting offset or mean exponent (*p*s>.10).

**Supplemental Table E - *Group by age effects for aperiodic parameters***

| Aperiodic parameter ~ | Regression Statistics | | |
| --- | --- | --- | --- |
| Model Predictors | *β (SE)* | *p* | *R^2^* |
| Exponent at Cz ~  Electrodes  Age  HR Group  LR Group  HR x Age  LR x Age | -.043 (.086)  **-.550 (.134)**  -.089 (.101)  .011 (.100)  **.260 (.121)**  .181 (.116) | .619  **<.001**  .377  .908  **.032**  .119 | .138 |
| Mean Exponent ~  Electrodes  Age  HR Group  LR Group  HR x Age  LR x Age | **-.191 (.093)**  **-.438 (.139)**  -.133 (.104)  -.005 (.104)  .204 (.125)  .161 (.120) | **.040**  **.002**  .200  .965  .103  .180 | .127 |
| Offset at Cz ~  Electrodes  Age  HR Group  LR Group  HR x Age  LR x Age | -.048 (.084)  **-.498 (.131)**  -.096 (.098)  .002 (.096)  .077 (.119)  .070 (.110) | .565  **<.001**  .328  .980  .516  .529 | .186 |
| Mean Offset ~  Electrodes  Age  HR Group  LR Group  HR x Age  LR x Age | -.084 (.092)  **-.451 (.135)**  -.177 (.101)  -.053 (.100)  .087 (.123)  .037 (.115) | .358  **.001**  .079  .596  .477  .748 | .170 |

*Note.* A series of regression models were conducted to assess predictors of aperiodic parameters and regression statistics are provided. Full Information Maximum Likelihood was used to handle missing data. HR Group = adolescents in HR group (coded 1) and other groups (coded 0). LR Group = adolescents in LR group (coded 1) and other groups (coded 0). Reference group = adolescents in the CD group.

**Supplemental Figure E1 – *Group by age for aperiodic parameters visualization***

Note. Scatter plots are provided for showing relationship between age and aperiodic activity for all groups. CD = Currently depressed. HR = High risk for depression due to maternal depression history. LR = Low risk for depression due to maternal depression history.
